# Targeting GGT1 Eliminates the Tumor-Promoting Effect and Enhanced Immunosuppressive Function of Myeloid-Derived Suppressor Cells Caused by G-CSF

**DOI:** 10.3389/fphar.2022.873792

**Published:** 2022-04-25

**Authors:** Zhiqi Xie, Takahiro Kawasaki, Haoyang Zhou, Daisuke Okuzaki, Naoki Okada, Masashi Tachibana

**Affiliations:** ^1^ Project for Vaccine and Immune Regulation, Graduate School of Pharmaceutical Sciences, Osaka University, Osaka, Japan; ^2^ Single Cell Genomics, Human Immunology, WPI Immunology Frontier Research Center, Osaka University, Osaka, Japan; ^3^ Genome Information Research Center, Research Institute for Microbial Diseases, Osaka University, Osaka, Japan; ^4^ Global Center for Medical Engineering and Informatics, Osaka University, Osaka, Japan

**Keywords:** myeloid-derived suppressor cells, granulocyte colony-stimulating factor, GGT1, febrile neutropenia, GGsTop

## Abstract

Myeloid-derived suppressor cells (MDSCs) are major immunosuppressive cells that accumulate in tumor-bearing hosts. Since MDSCs suppress anti-tumor immunity and promote tumor progression, they are promising targets for cancer immunotherapy. Granulocyte colony-stimulating factor (G-CSF) is an agent used for the treatment of chemotherapy-induced febrile neutropenia (FN) in patients with cancer. However, several reports have revealed that G-CSF plays crucial immune-related adverse roles in tumor progression through MDSCs. In this study, we showed that MDSCs differentiated in the presence of G-CSF *in vitro* exhibited enhanced proliferation and immunosuppressive activity compared to those differentiated without G-CSF. RNA sequencing analysis demonstrated that G-CSF enhanced the immunosuppressive function of MDSCs by upregulating gamma-glutamyltransferase (GGT) 1. Moreover, in the EL4 lymphoma-bearing neutropenic mouse model, administration of recombinant G-CSF increased the number of MDSCs and attenuated the anti-cancer effect of chemotherapy. We showed that the combination of GGsTop, a GGT inhibitor, could prevent G-CSF-induced tumor growth, without affecting the promotion of myelopoiesis by G-CSF. These results suggest that targeting GGT1 can mitigate G-CSF-induced enhanced immunosuppressive functions of MDSCs and can eliminate the tumor-promoting effect of G-CSF. Furthermore, GGsTop could be an attractive combination agent during G-CSF treatment for FN in patients with cancer.

## Introduction

Myeloid-derived suppressor cells (MDSCs) are immature myeloid cells that accumulate in cancer patients as well as in mouse tumor models. MDSCs play important roles in the suppression of anti-tumor immunity, resulting in the exacerbation of cancer (Fleming et al., 2018; Ostrand-Rosenberg and Fenselau, 2018; Veglia et al., 2021). Phenotypically and morphologically, MDSCs are classified into two subpopulations, namely monocytic (M)-MDSCs with CD11b^+^Ly-6G^−^Ly-6C^hi^ phenotype and polymorphonuclear (PMN)-MDSCs with CD11b^+^Ly-6G^+^Ly-6C^int^ phenotype (Bronte et al., 2016). M-MDSCs reportedly suppress T cell proliferation via arginase 1 and inducible nitric oxide synthase (iNOS), and PMN-MDSCs show an inhibitory effect via arginase 1 and reactive oxygen species (ROS) (Bronte et al., 2016). However, the mechanisms underlying the immunosuppressive function of MDSCs remain largely unexplored.

Febrile neutropenia (FN) is a deadly complication associated with cancer chemotherapy, usually indicating infection and sepsis, and commonly occurs following the initial cycles of myelosuppressive therapy (Klastersky et al., 2016). Prevention of FN reduces lengthy hospitalization, morbidity, mortality, and risk of chemotherapy reductions and delays (Kuderer et al., 2006; Mehta et al., 2015; Lucas et al., 2018). Granulocyte colony-stimulating factor (G-CSF) is a principal cytokine promoting and mobilizing granulocytes. Recombinant human G-CSF as prophylaxis and therapy has been shown to significantly reduce FN incidence and mortality following chemotherapy (Klastersky et al., 2016; Lucas et al., 2018). However, several observations suggest that G-CSF promotes tumor growth, metastasis, and resistance to chemotherapy by increasing the number of circulating MDSCs (Kawano et al., 2015; Pilatova et al., 2018) and is associated with a poor clinical prognosis (Hollmén et al., 2016; Liu et al., 2020). Although G-CSF has become the main therapeutic agent in cancer therapy for the treatment of neutropenia and prevention of FN, it is necessary to evaluate its impact on tumor-bearing patients. Therefore, considering the possible side effects of G-CSF in promoting tumor progression through MDSCs, there is an urgent need to elucidate the direct mechanism underlying the effect of G-CSF on MDSCs.

## Materials and Methods

### Mice

Female C57BL/6J mice were purchased from Japan SLC (Shizuoka, Japan). All mice were bred and maintained under specific pathogen-free conditions and used for experiments at 6–8 weeks of age. All experimental procedures in this study were performed in accordance with the institutional guidelines for animal experiments at the Osaka University, Japan.

### Cell Line

The EL4 cell lines were purchased from the American Type Culture Collection (ATCC) and maintained in RPMI-1640 medium (FUJIFILM Wako, Osaka, Japan) supplemented with 10% fetal bovine serum (FBS; Gibco, CA, United States) and 1% Antibiotic-Antimycotic Mixed Stock Solution (100 ×) (Nacalai Tesque, Kyoto, Japan). Cells were cultured according to the ATCC guidelines and used within 1 month of thawing from an early passage (<3 passages of original vial) lots.

### MDSC Differentiation *in vitro*

The *in vitro* differentiation of bone marrow (BM) cells into MDSCs was performed as described previously (Xie et al., 2018). Briefly, BM cells from C57BL/6J mice were stimulated with 40 ng/ml recombinant Granulocyte-macrophage CSF (GM-CSF) (Peprotech, NJ, United States) for 4 days in the absence or presence of G-CSF (5 ng/ml, BioLegend, CA, United States) and/or GGsTop (50 mΜ, FUJIFILM Wako) to examine their effects on MDSCs.

### Flow Cytometry Analysis

Cells were pelleted and washed in phosphate-buffered saline (PBS) supplemented with 2% FBS (2% FBS/PBS). The cell suspension was first blocked with TruStain fcX (anti-mouse CD16/32) antibody (BioLegend) for 5 min, and then stained with the following antibodies (Abs) for 15 min at 4°C: APC anti-mouse CD11b, Pacific Blue anti-mouse Gr-1, PE anti-mouse F4/80, FITC anti-mouse CD11c, APC-Cy7 anti-mouse Ly-6C, FITC anti-mouse Ly-6G, Pacific Blue anti-mouse CD4, and FITC anti-mouse CD8α (BioLegend). Next, the cells were washed and resuspended in 2% FBS/PBS. Shortly before performing measurements, a 7-amino actinomycin D viability staining solution (BioLegend) was added to each sample to stain dead cells. Flow cytometry analysis was performed on a BD FACSCanto II flow cytometer (BD Biosciences), and results were analyzed using the FlowJo software (version10.7.0, BD Biosciences).

### *In vitro* Suppression Assay

CD4^+^ T cells or CD8^+^ T cells were isolated from the spleens of C57BL/6J mice using the MojoSort magnetic cell separation system as previously described (Xie et al., 2018) and then labeled with the proliferation dye eFluor 670 (eBioscience, Thermo Fisher Scientific, CA, United States). The eFluor 670-labeled CD4^+^ T cells or CD8^+^ T cells were incubated with *in vitro* differentiated MDSCs at different ratios in a 96-well plate cultured with anti-mouse CD3ɛ Ab/anti-mouse CD28 Ab (BioLegend). After 3 days of incubation at 37°C in 5% CO_2_, the proliferation of CD4^+^ and CD8^+^ T cells determined by the eFluor 670 fluorescence intensity was analyzed using flow cytometry.

### Quantitative Reverse Transcription Polymerase Chain Reaction

PMN-MDSCs (Ly-6G^+^Ly-6C^int^) and M-MDSCs (Ly-6G^−^Ly-6C^hi^) were purified from *in vitro*-differentiated MDSCs cultured with or without the addition of G-CSF by fluorescence-activated cell sorting (purity >95%; JSAN, Bay bioscience Co., Ltd., Kobe, Japan). Total RNA was extracted from the *in vitro* differentiated MDSCs or sorted MDSC subsets and used to synthesize cDNA using the QuantiTect reverse transcription kit (Qiagen, Hilden, Germany) following the manufacturer’s instructions. qRT-PCR was performed using SYBR Premix Ex Taq (Tli RNaseH Plus; TaKaRa, Kusatsu, Japan) on a CFX96 touch real-time PCR detection system (Bio-Rad). The specific primer sequences are listed in Supplementary Table S1. Glyceraldehyde 3-phosphate dehydrogenase (*Gapdh*) was used as a reference gene, and relative expressions of other genes were calculated using the 2^−ΔΔCt^ method.

### RNA Sequencing Analysis

Total RNA was extracted from cells using the miRNeasy Mini kit (Qiagen) according to the manufacturer’s protocol. RNA libraries were prepared for sequencing using a TruSeq stranded mRNA sample prep kit (Illumina, CA, United States) according to the manufacturer’s instructions. Whole transcriptome sequencing was performed on RNA samples using the Illumina HiSeq 2500 platform in a 75 bp single-end mode. Volcano plot representation and pathway enrichment analysis of the differentially expressed genes were performed using BioJupies tools with adjusted *p* < 0.05 and fold change >2 (Torre et al., 2018). Raw data from this study have been submitted to Gene Expression Omnibus (GEO) under the accession number GSE183066.

### Gamma-Glutamyltransferase Activity Measurement

MDSCs (1 × 10^6^ cells) were homogenized in 200 µL of GGT assay buffer and centrifuged at 13,000 × *g* for 10 min following which the supernatant was collected. After reacting with the GGT substrate at 37°C for 40 min, fluorescence (365/460 nm) was measured using a microplate reader SpectraMax iD5 (Molecular Devices). GGT activity was calculated according to the protocol of the GGT Activity Colorimetric Assay Kit (BioVision, CA, United States).

### Glutamate Measurement

The *in vitro* MDSC culture medium was collected on day 4. Next, glutamate concentration was measured using Glutamate Assay Kit-WST (Dojindo, Kumamoto, Japan) according to the manufacturer’s protocol. Briefly, a glutamate standard or MDSC culture medium was added to a 96-well microplate; the working solution was then added to each well. After incubating the microplate at 37°C for 30 min, the absorbance was measured at 450 nm using SpectraMax iD5 (Molecular Devices). The glutamate concentration in each sample was calculated using a standard curve.

### ROS Level Measurement

Intracellular ROS was assayed using a ROS Assay Kit-Highly Sensitive DCFH-DA (Dojindo) according to the manufacturer’s protocol. Briefly, 2 × 10^5^ MDSCs were treated with 2′, 7′dichlorofluorescin diacetate (DCFH-DA) working solution and incubated for 30 min. Subsequently, the MDSCs were washed twice with Hanks’ Balanced Salt Solution, and ROS production was measured using a BD FACSCanto II flow cytometer (BD Biosciences).

### Cyclophosphamide -Induced Neutropenic Tumor Model

EL4 lymphoma cells (4 × 10^5^ cells/mouse) were injected subcutaneously into the lower right flank of C57BL/6J mice. Seven days after inoculation with EL4 cells, mice were intraperitoneally administered a single dose of CPA (100 mg/kg, FUJIFILM Wako) to create a neutropenic mouse model as described previously (Hattori et al., 1990). Starting from the same day, experimental mice received intraperitoneal injections of PBS, GGsTop (5 mg/kg, FUJIFILM Wako), and recombinant G-CSF (200 μg/kg, BioLegend) for six consecutive days. Before administration of the above reagent, mouse retro-orbital blood (approximately 75 μL) was collected for flow cytometry analysis and determining blood cell counts using a Sysmex XT-2000i automated hematology analyzer (Sysmex) daily. The tumor volume was calculated periodically up to day 15 using the following formula: Tumor Volume (cm^3^) = 0.5 × (Length × Width^2^).

### Statistical Analysis

Significant differences were assessed using Student’s *t*-test, or a one- or two-way analysis of variance (ANOVA) using GraphPad Prism 7.0 (GraphPad Software). Statistical significance was set at *p < 0.05*.

## Results

### G-CSF Regulates the Differentiation and Immunosuppressive Activity of MDSCs

**FIGURE 1:**
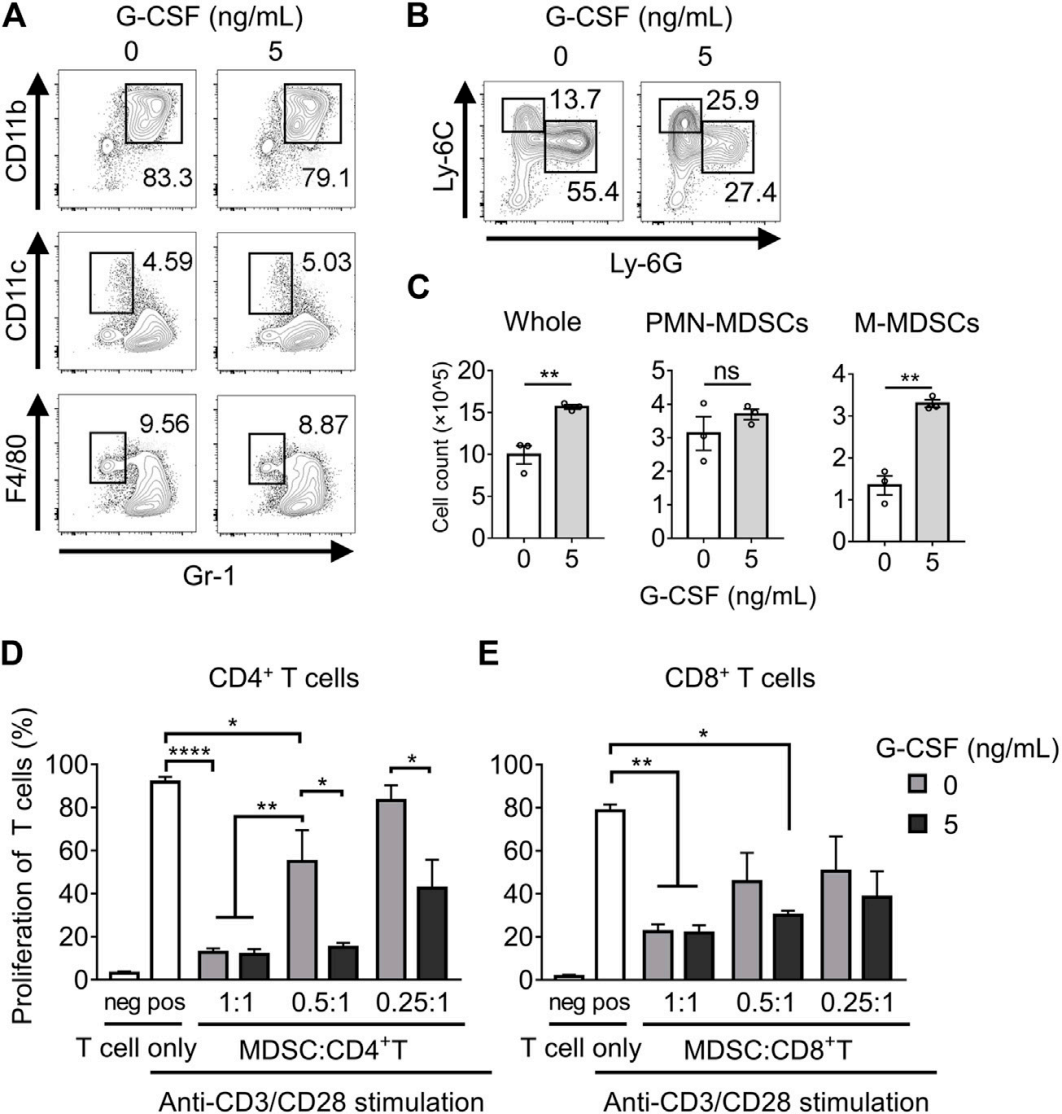
G-CSF regulates the differentiation and immunosuppressive activity of MDSCs. **(A)** Flow cytometry analysis of the percentage of MDSC (CD11b^+^Gr-1^+^), DC (Gr-1^−^CD11c^+^), and macrophage (Gr-1^-^F4/80^+^) populations after 4 days of culture in medium supplemented with GM-CSF (40 ng/ml) with or without G-CSF stimulation (5 ng/ml). **(B)** Flow cytometry analysis of the percentage of PMN-MDSCs (CD11b^+^Ly-6G^+^Ly-6C^int^) and M-MDSCs (CD11b^+^Ly-6G^−^Ly-6C^hi^) among CD11b^+^ cells. **(C)** Total numbers of *in vitro* MDSCs and their subset dyed with trypan blue are represented as means ± SEM (*n* = 3, Student’s *t*-test: **p < 0.01). **(D)** CD4^+^ or CD8^+^ T cells derived from WT mice were co-cultured with *in vitro* MDSCs under the stimulation of anti-CD3ɛ/CD28 antibodies. After three-day-culture, T cell proliferation in the absence or presence of MDSCs was examined by flow cytometry. Data are represented as means ± SEM (*n* = 5 pooled with two independent experiments, two-way ANOVA: *p < 0.05, **p < 0.01, and ****p < 0.0001).

Previous studies have reported that GM-CSF stimulates BM cells to differentiate into MDSCs (hereafter referred to as *in vitro* MDSCs). Freshly isolated BM cells were cultured for 4 days in a medium supplemented with or without G-CSF and GM-CSF to examine whether G-CSF affects the differentiation of *in vitro* MDSCs. G-CSF did not affect the percentage of MDSCs (CD11b^+^Gr-1^+^), dendritic cells (DCs; Gr-1^−^CD11c^+^), or macrophages (Gr-1^-^F4/80^+^) differentiated from BM cells, as shown in Figure 1A. The proportion of M-MDSCs with the CD11b^+^Ly-6G^−^Ly-6C^hi^ phenotype increased in the presence of G-CSF, whereas PMN-MDSCs with the CD11b^+^Ly-6G^+^Ly-6C^int^ phenotype decreased (Figure 1B). However, since whole BM cells largely increased with stimulation by G-CSF, there was no change in the number of PMN-MDSCs, and M-MDSCs substantially increased (Figure 1C). These results suggest that G-CSF promotes the proliferation of MDSCs, especially M-MDSCs.

Next, we investigated whether G-CSF affected the immunosuppressive activity of *in vitro* MDSCs. Suppression of CD4^+^ T cell proliferation was observed in *in vitro* MDSCs that were not treated with G-CSF, and the immunosuppressive activity was impaired with decreasing numbers of MDSCs. Moreover, the immunosuppressive activity of *in vitro* MDSCs was significantly enhanced by G-CSF treatment (Figure 1D). Similar results were obtained in the CD8^+^ T cell suppression assay (Figure 1E). These observations revealed that T cells, especially CD4^+^ T cells, were less proliferative when co-cultured with G-CSF-conditioned *in vitro* MDSCs, suggesting that G-CSF strongly enhanced the immunosuppressive activity of *in vitro* MDSCs.

### Identifying G-CSF-Induced MDSC Gene Expression Profile Using RNA-Seq

**FIGURE 2:**
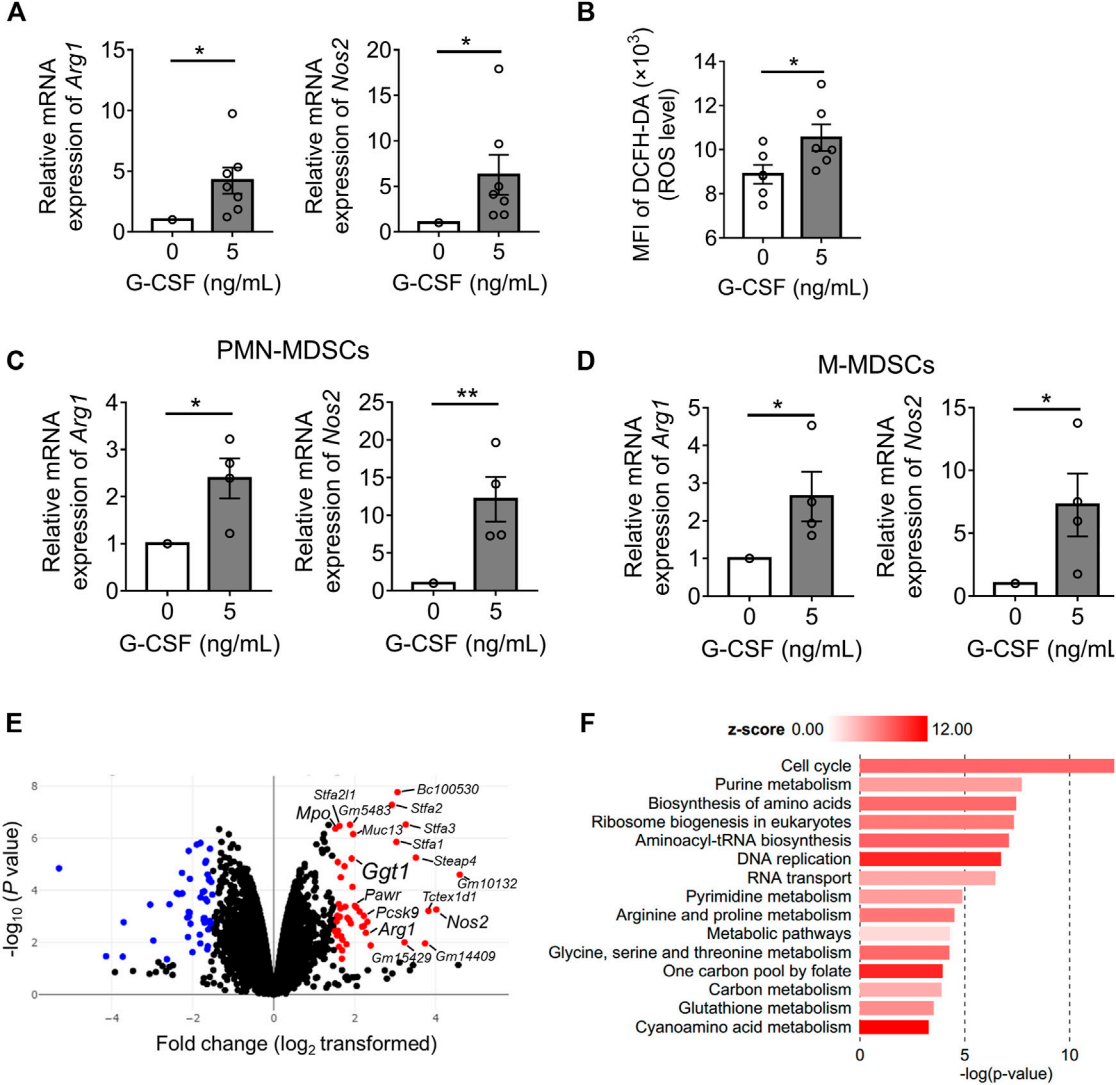
Identifying G-CSF-induced MDSC gene expression profile using by RNA sequencing. **(A)** The mRNA expression of *Arg1* and *Nos2* of *in vitro* MDSCs cultured with GM-CSF with or without the addition of G-CSF was measured by qRT-PCR. Data are represented as means ± SEM of three independent experiments and normalized to the expression of *Gapdh* housekeeping gene (*Arg1*: *n* = 7; *Nos2*: *n* = 7; Student’s *t*-test: *p < 0.05). **(B)** Intracellular ROS level was measured by flow cytometry analysis using DCFH-DA (means ± SEM, *n* = 4 pooled with two independent experiments, Student’s *t*-test: *p < 0.05). **(C,D)** The mRNA expressions of *Arg1* and *Nos2* in the sorted PMN-MDSCs and M-MDSCs were measured by qRT-PCR (means ± SEM, *n* = 4 pooled with two independent experiments, Student’s *t*-test: *p < 0.05, **p < 0.01). **(E)** Volcano plots showing differences in the mRNA expression profile of *in vitro* MDSCs (*n* = 2 biological replicates). **(F)** KEGG pathway enrichment analysis of significantly upregulated gene sets in MDSCs stimulated by G-CSF using BioJupies tools (*n* = 2 biological replicates).

To explore the mechanism by which G-CSF enhances the immunosuppressive function of MDSCs, we first analyzed the expression of the main immunosuppressive molecules of MDSCs: arginase 1 and iNOS as well as ROS by qRT-PCR or flow cytometry analysis. We found that the mRNA levels of *Arg1* and *Nos2* were significantly upregulated by G-CSF and that ROS levels determined by DCFH-DA also increased (Figures 2A,B). Besides, G-CSF upregulated the expression of *Arg1* and *Nos2* in both PMN-MDSCs and M-MDSCs, indicating that the G-CSF-induced upregulation of those genes was because of the enhanced transcription of the genes but not because of the alteration of the population of MDSC subsets (Figures 2C,D).

Next, we performed an RNA-seq analysis, comparing the gene expression profiles of *in vitro* MDSCs differentiated with or without G-CSF. There were 228 genes differentially expressed with fold change >2 and adjusted *p* < 0.05 when comparing both groups. Among these genes, 89 genes were expressed at higher levels in MDSCs differentiated in the presence of G-CSF than in those differentiated without G-CSF, and 139 genes showed lower expression. Increased expression of *Arg1*, *Nos2*, and *Mpo*, which are the immunosuppressive molecules of MDSCs, was observed (Figure 2E), consistent with the above results. Additionally, pathway enrichment analysis revealed that the MDSCs differentiated with G-CSF displayed the activation of the cell cycle, metabolism, and DNA replication, which would account for the enhanced proliferation of MDSCs differentiated with G-CSF (Figure 2F).

### GGT1 Is Involved in the Enhanced Immunosuppressive Function of MDSCs by G-CSF

Next, among the top 20 upregulated genes by G-CSF, genes associated with poor prognosis (Logrank *p* < 0.05) in more than four cancers, including *Nos2*, proprotein convertase subtilisin/kexin type 9 (*Pcsk9*), PRKC apoptosis WT1 regulator (*Pawr*), and gamma-glutamyltransferase 1 (*Ggt1*), were identified as candidate factors involved in the immunosuppressive ability of MDSCs using the GEPIA web server (Tang et al., 2017) (Supplementary Figure S1). GGT1 is a key enzyme involved in several metabolic processes, such as glutathione metabolism and cyanoamino acid metabolism (Verma et al., 2015), which were also enriched and active in MDSCs differentiated with G-CSF (Figure 2F). Furthermore, GGT is expressed on the cell surface, acts as a glutathione hydrolase that increases extracellular cysteinyl glycine and glutamate, and is reported to be associated with the progression of several tumors (Wang et al., 2017; Ince et al., 2020). Thus, to identify novel factors involved in the immunosuppressive ability of MDSCs, we focused on the *Ggt1* gene.

**FIGURE 3:**
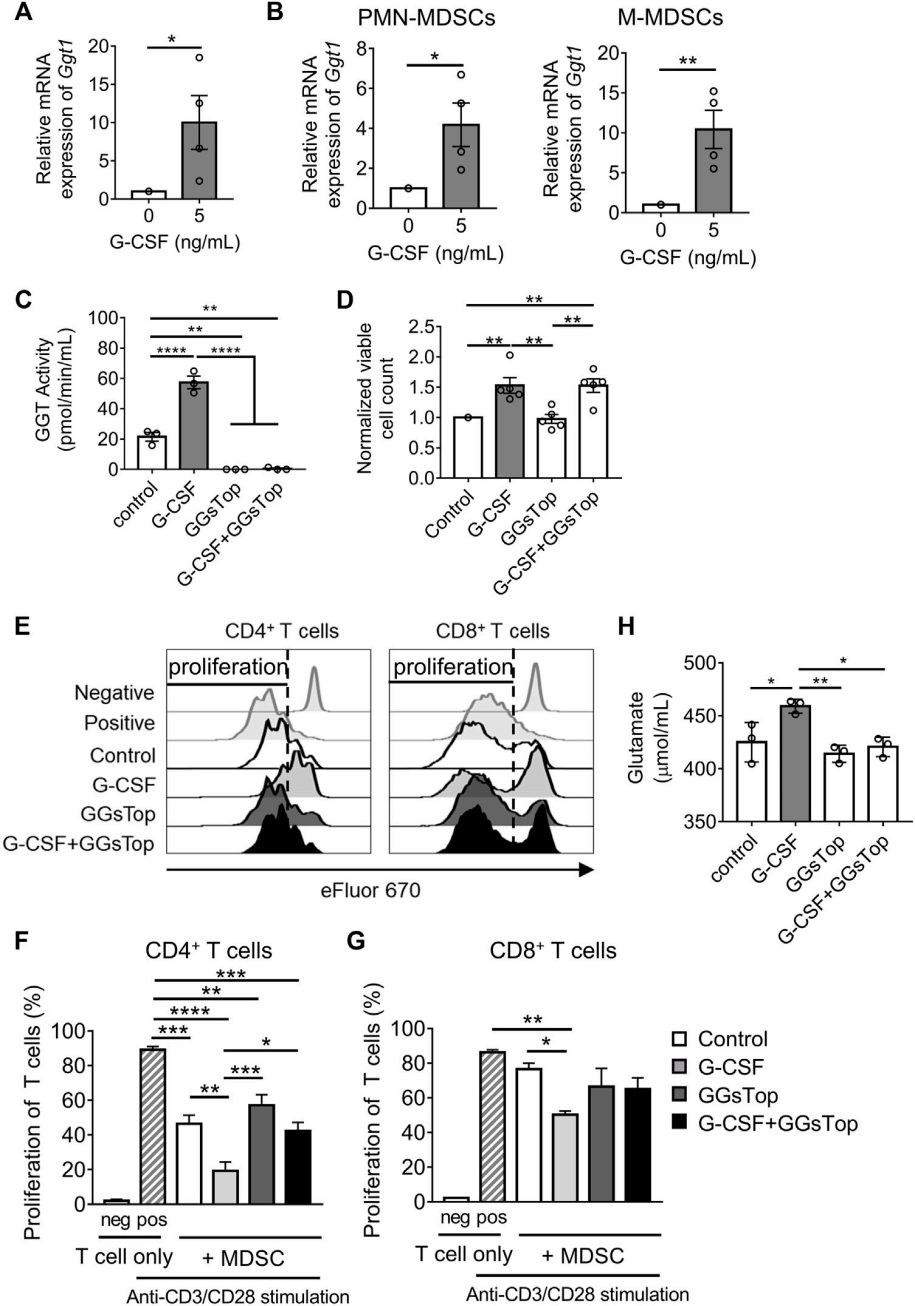
GGT1 is involved in the G-CSF-enhanced immunosuppressive function of MDSCs. **(A)** The mRNA expression of *Ggt1* in *in vitro* MDSCs was measured by qRT-PCR. Data are represented as means ± SEM of three independent experiments (*n* = 4, Student’s *t*-test: *p < 0.05). **(B)** The mRNA expression of *Ggt1* in the sorted PMN-MDSCs and M-MDSCs was measured by qRT-PCR (means ± S.E.M., *n* = 4 pooled with two independent experiments, Student’s *t*-test: *p < 0.05, **p < 0.01). **(C)** GGT activity of *in vitro* MDSCs cultured with or without the addition of G-CSF and/or GGsTop was measured using a GGT activity assay kit (means ± SEM, *n* = 4 pooled with two independent experiments, one-way ANOVA: *p < 0.05, **p < 0.01, and ****p < 0.0001). **(D)** The numbers of *in vitro* MDSCs were measured by trypan blue dye exclusion method (means ± SEM, *n* = 5 pooled with three independent experiments, one-way ANOVA: **p < 0.01). **(E–G)** MDSCs conditioned with or without G-CSF or GGsTop were combined in a 0.5:1 ratio with eFluor 670-labeled CD4^+^ or CD8^+^ T cells, followed by stimulation with anti-CD3ɛ/CD28 antibodies. **(E)** Representative histograms of eFluor 670 expressions in CD4^+^ or CD8^+^ T cells. **(F,G)** T cell proliferation was examined by flow cytometry. Data are represented as means ± SEM (two-way ANOVA: *p < 0.05, **p < 0.01, ***p < 0.001, and ****p < 0.0001). **(H)** The glutamate level was measured from the culture medium of *in vitro* MDSCs by glutamate assay-kit (means ± SEM, *n* = 4 pooled with two independent experiments, one-way ANOVA: *p < 0.05, **p < 0.01).

The upregulation of the mRNA of *Ggt1* by G-CSF in MDSCs was verified by qRT-PCR (Figure 3A). Besides, *Ggt1* mRNA expression was more upregulated in M-MDSCs than in PMN-MDSCs upon G-CSF stimulation, suggesting that M-MDSCs can play an important role in increasing the G-CSF-promoted immunosuppressive effects (Figure 3B). To elucidate whether GGT1 is involved in the enhanced immunosuppressive function of MDSCs by G-CSF, we used GGsTop, a highly selective GGT inhibitor. The GGT activity of MDSCs was enhanced by G-CSF, which was inhibited below the detection limit in the GGsTop alone and G-CSF/GGsTop combination groups (Figure 3C). There was no change in the number of MDSCs in the presence of GGsTop (Figure 3D), suggesting that the inhibition of GGT did not affect the G-CSF-induced proliferation of BM cells. Meanwhile, the addition of GGsTop did not affect the proportion and number of M-MDSCs or PMN-MDSCs (Supplementary Figures S2A,B). We further investigated whether GGsTop cancels the enhancement of the immunosuppressive activity of *in vitro* MDSCs by G-CSF. Consistent with *Ggt1* expression, the immunosuppressive activity of *in vitro* MDSCs was significantly increased by G-CSF, while the combination of GGsTop and G-CSF attenuated the immunosuppressive activity to the same level as that in the control group (Figures 3E–G). Additionally, GGsTop alone did not affect the immunosuppressive activity of *in vitro* MDSCs. Furthermore, we examined the effect of GGsTop alone or the G-CSF/GGsTop combination on *Arg1*, *Nos2*, and ROS expression. The G-CSF/GGsTop combination group downregulated the expression of *Arg1* and *Nos2*. Unexpectedly, GGsTop alone upregulated their expression (Supplementary Figure S2C). As *Arg1* and *Nos2* upregulation by GGsTop alone did not increase the immunosuppressive ability of MDSCs (Figures 3E,F), suggesting that the upregulation of *Arg1* and *Nos2* did not play a decisive role in increasing the MDSC immunosuppressive ability caused by G-CSF. In addition, GGsTop did not abrogate the expression of ROS caused by G-CSF, suggesting that ROS is not a key factor either (Supplementary Figure S2D). These results suggest that inhibition of GGT1 activity eliminates G-CSF-induced enhanced immunosuppressive function of MDSCs, but has no effect on its ability to promote proliferation.

The key role of GGT1 is to hydrolyze glutathione. We speculated that metabolites of glutathione, such as glutamate and cysteinyl glycine, obtained by the action of GGT1, enhance the immunosuppressive function of MDSCs. Elevated glutamate concentrations are commonly observed in tumor patients, and studies indicate that glutamate could regulate the immunosuppressive activity of MDSCs on the proliferation of T cells (Morikawa et al., 2018; Wu et al., 2019). Therefore, we measured the concentration of glutamate to determine whether the level of glutamate is regulated by GGT1. Glutamate levels were increased on treating cells with G-CSF and were decreased on treatment with GGsTop both alone and in combination with G-CSF (Figure 3H). GGsTop alone or the G-CSF/GGsTop combination did not significantly reduce glutamate levels compared with the control, which explained why GGsTop alone did not inhibit MDSC-mediated T cell suppression. These observations revealed that elevated GGT activity induced by G-CSF increased glutamate levels, and that GGT1 is a novel factor involved in the enhanced immunosuppressive function of MDSCs induced by G-CSF.

### GGsTop Eliminates the Effect of G-CSF in Promoting Tumor Growth

**FIGURE 4:**
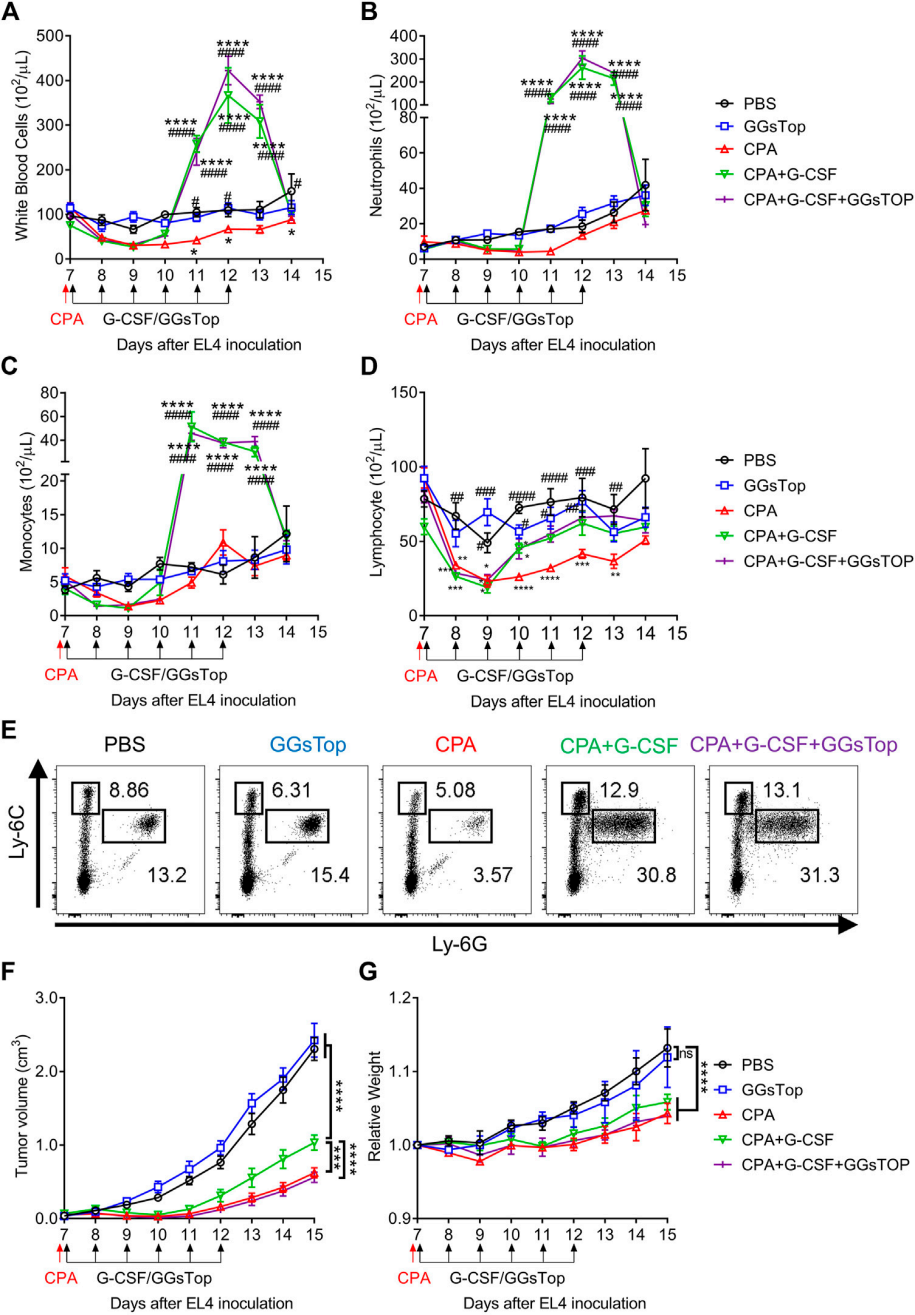
GGsTop eliminates the effect of G-CSF in promoting tumor growth. **(A–D)** Dynamics of circulating **(A)** white blood cells, **(B)** neutrophils, **(C)** monocytes, and **(D)** lymphocytes was examined using a Sysmex XT-2000i automated hematology analyzer (means ± SEM, pooled from two independent experiments with *n* = 6, *p < 0.05, **p < 0.01, ***p < 0.001 and ****p < 0.0001 compared with PBS group by two-way ANOVA; ^#^ *p* < 0.05, ^##^ *p* < 0.01, ^###^ *p* < 0.001 and ^####^ *p* < 0.0001 compared with CPA group by two-way ANOVA). **(E)** Flow cytometric analysis of MDSC subsets in the blood at 4 days after CPA/G-CSF/GGsTop administration. **(F,G)** Tumor volumes and relative body weight were calculated periodically and are shown as means ± SEM, pooled from two independent experiments with *n* = 6 (***p < 0.001, and ****p < 0.0001 by two-way ANOVA).

Given that G-CSF enhances the immunosuppressive function and proliferation of MDSCs, it is possible that G-CSF causes side effects through MDSCs when used to prevent FN. We then tested whether G-CSF promoted MDSCs and tumor progression using the EL4 lymphoma-bearing neutropenic mouse model. In the EL4 tumor-bearing mouse model, administration of the anti-tumor drug CPA reduced white blood cells, including neutrophils, monocytes, and lymphocytes to 50% below the pretreatment value measured using an XT-2000i hematology analyzer, leading to neutropenia (Figures 4A–D). CPA strongly inhibited tumor growth (Figure 4F). In contrast, the accelerated recovery of white blood cells was observed in mice administered G-CSF after CPA. G-CSF dramatically increased neutrophil and monocyte counts from days 11–13 and facilitated the recovery of lymphocytes to control levels (Figures 4A–D), which would reduce the risk associated with CPA-induced neutropenia. Flow cytometry analysis revealed that the proportion of M-MDSCs and PMN-MDSCs in the G-CSF group increased significantly, suggesting that G-CSF promoted neutrophils and monocytes that were morphologically identified using an XT-2000i hematology analyzer to be mixed with most MDSCs (Figure 4E). Furthermore, tumor progression was observed after G-CSF administration, suggesting that G-CSF-induced MDSCs attenuated the anti-tumor effect of CPA (Figure 4F). Next, we explored whether GGsTop could inhibit the growth of tumors induced by G-CSF while maintaining myelopoiesis by G-CSF. As expected, GGsTop alone did not affect the dynamics of myelopoiesis (Figures 4A–E) and tumor progression (Figure 4F). The combination of GGsTop and G-CSF promoted the proliferation of white blood cells, neutrophils, and monocytes in the G-CSF group and inhibited tumor growth in the presence of CPA. Consistent with other studies (Jounaidi and Waxman, 2001; Kim et al., 2018), due to CPA drug toxicity and rapid tumor growth in the PBS and GGsTop groups, the biological significance of body weight was shown in the CPA-treated and control mice. Moreover, the results showed that GGsTop was well-tolerated, with no significant decrease observed in the body weight (Figure 4G). These results suggest that targeting GGT eliminates the effect of G-CSF in promoting tumor growth. GGsTop could be an attractive combination agent with G-CSF for the treatment of neutropenia in patients with cancer.

## Discussion

To date, G-CSF administration is commonly used in the management of cancer patients and dramatically reduces the risks associated with chemotherapy-induced FN (Pilatova et al., 2018). However, an increasing number of studies have found that G-CSF enhances the pro-tumor effects of MDSCs, thereby leading to poor prognosis and chemoresistance in cancers (Hollmén et al., 2016; Liu et al., 2020; Karagiannidis et al., 2021). Therefore, the safety of G-CSF as an adjunct to cancer treatment should be addressed. Although further research is needed, our pre-clinical data showed that G-CSF promotes tumor growth in the CPA-induced neutropenia EL4 tumor-bearing model, which would result from the enhancement of immunosuppressive function and the increase in the number of MDSCs by G-CSF.

To eliminate the side effects of G-CSF, it is necessary to elucidate the direct mechanism underlying the action of G-CSF on MDSCs. We utilized *in vitro* MDSCs and RNA-seq analysis and identified that GGT1 played a pivotal role in enhancing the immunosuppressive function of MDSCs by G-CSF. GGT1 is a member of the GGT family, which also includes other proteins, such as GGT2, GGT3P, GGT4P, GGT5, GGT6, GGT7, and GGT8P. Among these, GGT1 and GGT5 are the only two enzymes with catalytic activity (Takemura et al., 2021), and the catalytic rate of GGT1 is approximately 46 times faster than that of GGT5 (Wickham et al., 2011). Therefore, GGT1 is the main catalytically active enzyme in the GGT family. It has been reported that the activity of GGT is increased in cancers resistant to chemotherapy, and the removal of glutathione, the substrate of GGT, in tumors inhibits tumor progression and metastasis (Mena et al., 2007; Hanigan, 2014). Our results also showed that G-CSF upregulated GGT1 expression on MDSCs, while GGsTop canceled the enhanced T cell proliferation inhibitory ability of MDSCs induced by G-CSF. Additionally, we showed that G-CSF increased the level of glutamate in the culture medium. GGT1 hydrolyzes extracellular glutathione to produce glutamate. Glutamate was originally discovered to be the main excitatory neurotransmitter in the central nervous system and can also act as a signaling molecule in other tissues (Affaticati et al., 2011). Recent studies have shown that glutamate could promote the activation of early MDSCs and the high concentrations of extracellular glutamate can suppress T cell activation (Poulopoulou et al., 2005; Wu et al., 2019). Previously, we found that glutamate signaling through metabotropic glutamate receptor 2/3 increases the potency of the immunosuppressive ability of MDSCs, especially the inhibition of CD4^+^ T cell proliferation, similar to G-CSF (Morikawa et al., 2018). The expression of glutamate receptors differs among different types of T lymphocytes (Pacheco et al., 2007); thus, CD8^+^ T cells may express lower glutamate receptor levels, resulting in the relative resistance of CD8^+^ T cells to the increased suppressive function of MDSCs cultured with G-CSF. These results indicate that the mechanism by which G-CSF enhances the immunosuppressive ability of MDSCs may involve GGT-induced increased glutamate levels.

GGsTop is now commercially available and its beneficial properties, such as being non-toxic, highly selective, and potent irreversible inhibitor of GGT activity, have been reported under a variety of experimental conditions (Kamiyama et al., 2016). For example, it has been reported that GGsTop could quickly and safely treat oral mucositis induced by cancer chemotherapy (Shimamura et al., 2019), reduce hepatic ischemia-reperfusion injury (Tamura et al., 2016), and may protect against chronic experimental autoimmune encephalomyelitis progression (Mendiola et al., 2020). Our results demonstrated its safety and effectiveness in an *in vivo* CPA-induced neutropenic tumor model, indicating that GGsTop can eliminate the effect of G-CSF in promoting tumor growth. Inhibition of GGT1 by GGsTop did not affect the viability and proliferation of MDSCs *in vitro,* as assessed by cell number quantification. The numbers of white blood cells, neutrophils, monocytes, and lymphocytes were similar after 6 days of treatment with GGsTop compared to PBS control *in vivo*. Accordingly, the inhibition of GGT1 by another inhibitor, acivicin, also showed no difference in microglia, macrophages, and whole blood cell numbers, but altered the glutathione metabolism of those cells (Mendiola et al., 2020). These results indicated that inhibiting GGT1 regulated the metabolism and activity of myeloid cells, including MDSCs, but had no effect on their proliferative activity.

In conclusion, our study showed that G-CSF directly enhanced the proliferation and immunosuppressive function of MDSCs and identified GGT1 as a novel factor involved in the enhanced immunosuppressive function of MDSCs induced by G-CSF. Furthermore, our results demonstrated that tumor progression in the chemotherapy-induced neutropenic mouse model was promoted by G-CSF, and the specific GGT inhibitor, GGsTop, prevented G-CSF-induced EL4 lymphoma progression. Although our findings suggest that GGsTop exerts its effects by targeting GGT1 on MDSCs, GGT1 is also expressed in a variety of cells other than MDSCs, and the possibility that GGsTop functions through other cellular mechanisms cannot be excluded, thus suggesting the requirement for further studies. Our study also still lacks clinical human experimental data to prove the safety and efficacy of GGsTop, the existing results suggest that GGsTop would be effective in future clinical applications. These findings should contribute to the development of safer and more effective treatment strategies for FN in patients with cancer.

## Data Availability

The datasets presented in this study can be found in online repositories. The names of the repository/repositories and accession number(s) can be found in the article/Supplementary Material.
