# Supplementary material for: Targeting GGT1 Eliminates the Tumor-Promoting Effect and Enhanced Immunosuppressive Function of Myeloid-Derived Suppressor Cells Caused by G-CSF
Source: Front Pharmacol. 2022 Apr 25;13:873792. doi: 10.3389/fphar.2022.873792 (PMC9081766; doi:10.3389/fphar.2022.873792)
Supplement: Supplementary file 1 [file DataSheet1.pdf]

Supplementary Material

Table 1. List of primer sequences used in qRT-PCR analysis.

Gene	Forward primer	Reverse primer
Gapdh	TGACCTCAACTACATGGTCTACA	CCGTGAGTGGAGTCATACTGG
Arg1	CCTATGTGTCATTTTGGGTGGATG	GGTTGTCAGGGGAGTGTTGAT
Nos2	GGAGTGACGGCAAACATGACT	TAGCCAGCGTACCGGATGA
Ggt1	CGCAAGCCTGCTGTGTATG	CGGGCATTGATAACCTCAACTT

Supplementary Figure 1. Survival curves comparing the high (red line) and low (blue line) (50% cut-off) expression of *Nos2*, *Pcsk9*, *Pawr*, and *Ggt1* in distinct types of cancers with $p < 0.05$ by the Log-rank test in the GEPIA web server. ACC, adrenocortical carcinoma; BLCA, bladder urothelial carcinoma; BRCA, breast invasive carcinoma; GBM, glioblastoma multiforme; KIRC, kidney renal clear cell carcinoma; KIRP, kidney renal papillary cell carcinoma; LAML, acute myeloid leukemia; LGG, brain lower grade glioma; LIHC, liver hepatocellular carcinoma; LUAD, lung adenocarcinoma; LUSC, lung squamous cell carcinoma; MESO, mesothelioma; PAAD, pancreatic adenocarcinoma; SKCM, skin cutaneous melanoma; UVM, uveal melanoma.

Supplementary Figure 2. A, B) The number of PMN-MDSCs (CD11b^{+}Ly-6G^{+}Ly-6C^{int}) and M-MDSCs (CD11b^{+}Ly-6G^{-}Ly-6C^{hi}) are represented as mean ± S.E.M. ($n = 4$ pooled with two independent experiments, one-way ANOVA: *$p < 0.05$, **$p < 0.01$). **C)** The mRNA expression of *Arg1* and *Nos2* in *in vitro* MDSCs cultured with or without the addition of G-CSF or GGsTop were measured by qRT-PCR. Data were normalized to the expression of *Gapdh* housekeeping gene and compared to mRNA expression in control MDSCs (means ± S.E.M., $n = 4$ pooled with three independent experiment, one-way ANOVA: *$p < 0.05$, **$p < 0.01$, ***$p < 0.001$, ****$p < 0.0001$). **D)** Intracellular ROS level was measured by flow cytometry analysis using DCFH-DA (means ± S.E.M., $n = 4$ pooled with two independent experiments, one-way ANOVA: *$p < 0.05$, **$p < 0.01$).

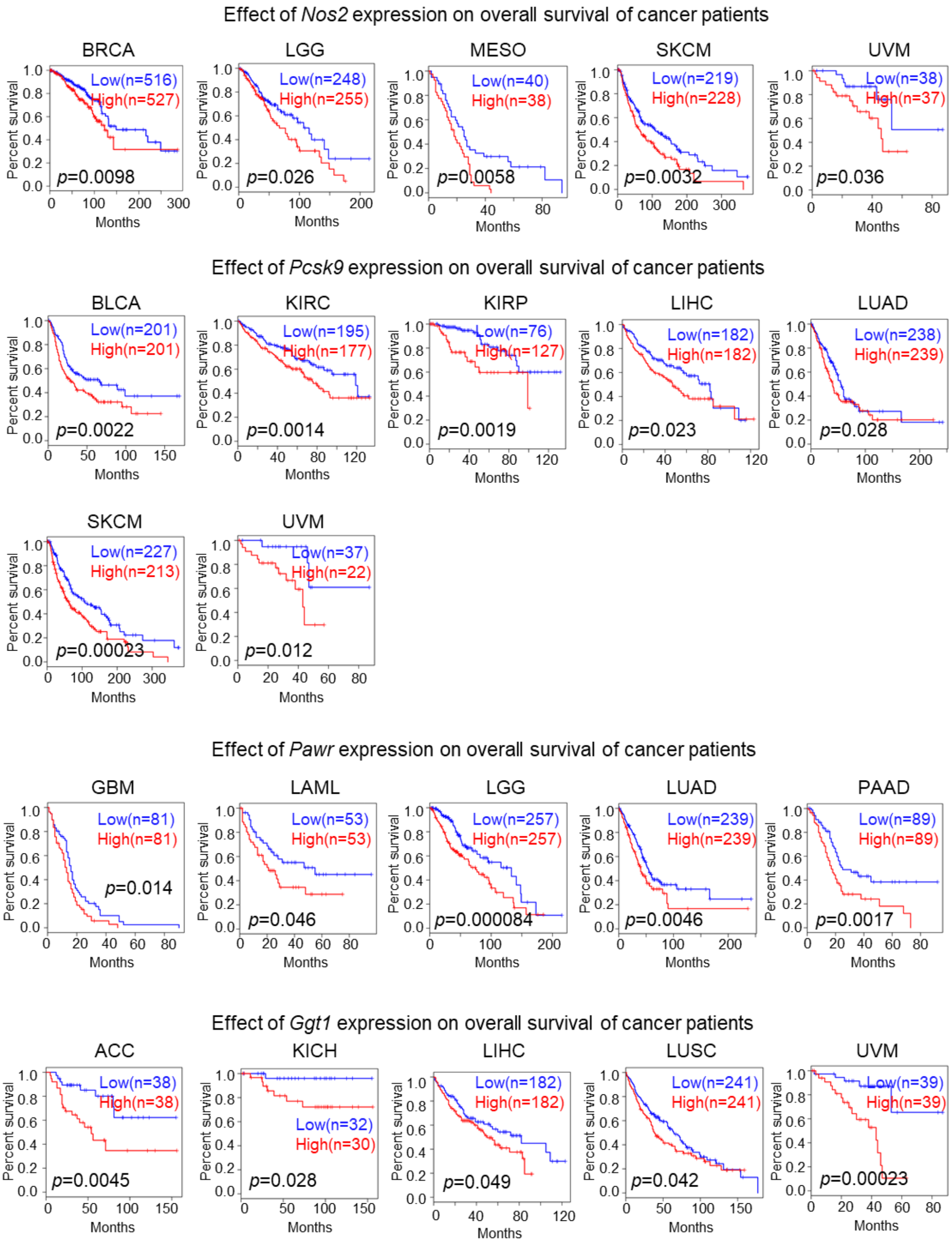

Supplementary Figure 1

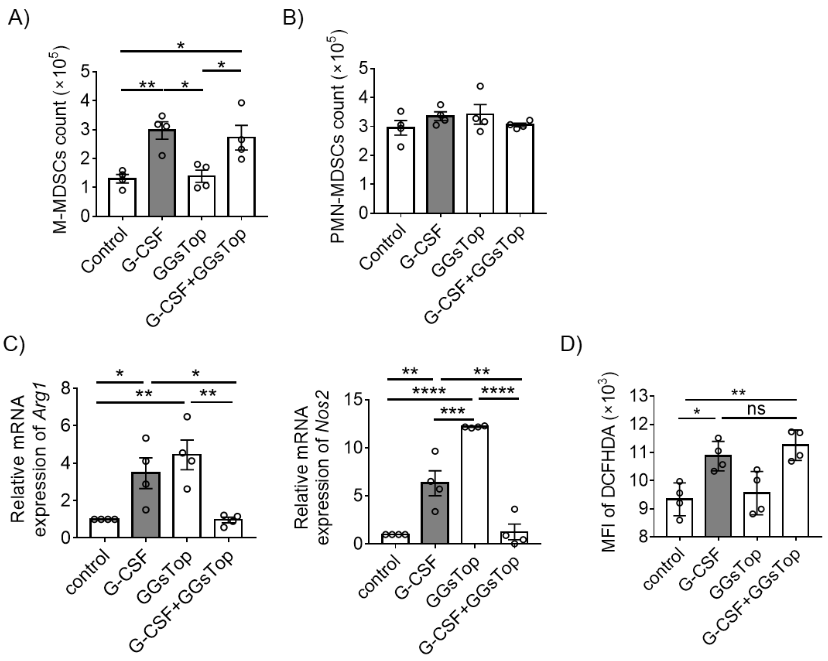

Supplementary Figure 2
